# Synthesis and Fluorescent Property Study of Novel 1,8-Naphthalimide-Based Chemosensors

**DOI:** 10.3390/molecules23020376

**Published:** 2018-02-10

**Authors:** Ying Fu, Xiao-Xiao Pang, Zhi-Qiang Wang, Hai-Tao Qu, Fei Ye

**Affiliations:** 1 Department of Applied Chemistry, College of Science, Northeast Agricultural University, Harbin 150030, China; fuying@neau.edu.cn (Y.F.); pangxiaoxiaoyk@163.com (X.-X.P.); wzq19910101@163.com (Z.-Q.W.); 2 Quality supervision and Inspection Institute, Harbin 150030, China; Handsome.qu@gmail.com

**Keywords:** *N*-n-Butyl-4-(*N*′,*N*′-dihydroxyethylamino)-1,8-naphthalimide, design, synthesis, crystal structure, fluorescence

## Abstract

A series of novel mono- and di-substituted *N*-n-butyl-1,8-naphthalimide derivatives were synthesized simultaneously via a three-step reaction. The single crystal structure of *N*-n-butyl-4-[*N*′,*N*′-bis(2′,4′-dichlorobenzoyl)ethylamino]-1,8-naphthalimide (**3f**) was determined. The UV-vis and fluorescence properties of compound **3f** were investigated. The **3f** showed highly selective and sensitive fluorescence changes response towards Pb^2+^. A titration of monomer with Pb^2+^ ion was performed. When Pb^2+^ ion concentration increased from 0 to 10 eq., the fluorescent intensity of **3f** decreased from 199.97 to 48.21. The pH effect on **3f** showed that it is stable in a wide range of pH. The results indicated that **3f** might be a probe molecule for Pb^2+^.

## 1. Introduction

Determining the contents of metal cations in environmental objects and biological systems is an important practical task for industry, medicine and ecology and for chemical and biochemical studies [1,2,3,4,5]. Among the extensive modern physicochemical methods of analysis, optical electron spectroscopy has gained great popularity due to the relative simplicity of experimental procedures combined with its high sensitivity toward analytes. In particular, fluorescent chemosensors have been proven to be highly valuable tools for sensing chemical and biological species such as metal ions, anions and amino acids, because of their high sensitivity, selectivity, rapidity, portability and the availability of a wide range of indicator dyes, etc. [6]. There is no doubt that fluorescent chemosensors with differential responses toward multiple components are economic and highly desirable for practical applications.

In recent years, 1,8-naphthalimide derivatives have been widely applied as fluorescent dyes, metal sensors and organic light emitting materials because of their excellent fluorescence properties, high absorption coefficients, good fluorescence and quantum yields, large stokes shifts, good stability and easy modification. The considerable interest in 1,8-naphthalimide derivatives as the photoactive component of optical chemosensors is caused, on the one hand, by relative simplicity of their synthesis, on the other hand, by the great diversity of their photo-physical properties [7,8,9]. Naphthalimide-based probes have been developed to detect H^+^, Hg^2+^, Zn^2+^, Cu^2+^, Ag^+^, Cd^2+^, Pd^2+^, Cr^3+^, Al^3+^, Fe^3+^ and F^−^ via chromogenic and fluorometric analyses [10,11,12,13,14,15]. Therefore, the naphthalimide derivatives are potential carriers that could be used in the preparation of new optical chemosensors. Though extensive excellent work has been reported on the 1,8-naphthalimide derivatives with an *O-* [16,17], *N-* [18], *S-*substituted group [19], or five-member heterocycle [20,21] in the ***C***-4 position as an electron donating group, it is still meaningful to extend the research of such materials, including introducing different electron-donating groups, such as hydroxyl or ester group to explore their fluorescence property. However, few studies have focused on fluorescent 1,8-naphthalimide esters containing mono- or di-substituted ester at the ***C***-4 position.

**Scheme 1:**
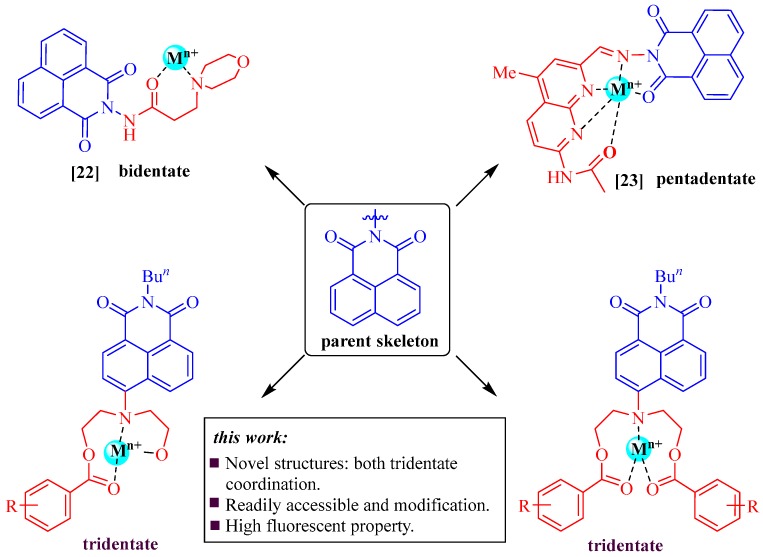
Design of the novel 1,8-naphthalimide-based chemosensors.

In this study, we designed and synthesized a series of *N*-n-butyl-1,8-naphthalimide fluorescent sensors with a diethyl amino link at the ***C***-4 position. As shown in Scheme 1, a functional group containing a nitrogen atom and a carbonyl group was introduced to 1,8-naphthalimide; these groups are commonly used as acceptor units in probes designed for metal ions and anions [22,23]. The biological importance of the combination of the carbonyl groups with naphthalimide prompted us to explore a new class of fluorescent probe for selective recognition of metal ions in aqueous media. This is the first attempt to synthesize mono- and di-substituted 1,8-naphthalimide esters simultaneously at the ***C***-4 position. In addition, a single crystal was prepared to characterize its spatial configuration and to propose its possible complex with metal ions. The spectroscopic changes that result from structural changes signal the existence of different metal ions.

## 2. Results and Discussion

### 2.1. Synthesis and Characterization

**Scheme 2:**
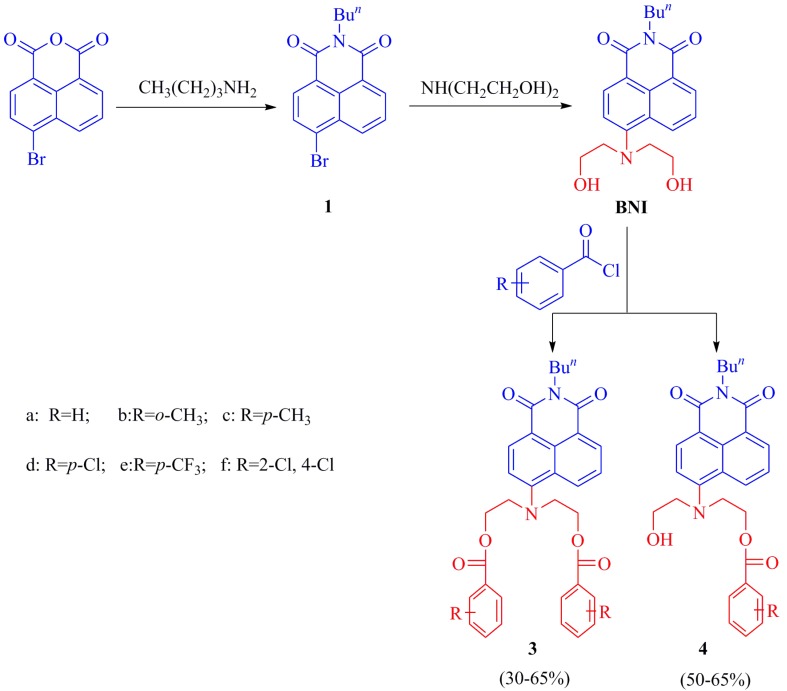
Synthetic route for the production of compounds **3** and **4**.

As shown in Scheme 2, the mono- and di-substituted 1,8-naphthalimide esters were synthesized via three steps. Although many efforts have been made to prepare 1,8-naphthalimide derivatives with different substitutions at position 4 [1,9,10,24,25,26,27,28], this is the first attempt to synthesize mono- and di-substituted 1,8-naphthalimide esters simultaneously at position 4. *N*-n-Butyl-4-bromo-1,8-naphthalimide was prepared by the convenient substitution of 4-bromo-1,8-naphtalic anhydride with n-butyl amine. The intermediate *N*-n-butyl-4-(*N*′,*N*′-dihydroxyethyl)amino-1,8-naphthalene imide (**BNI**) was obtained through nucleophilic substitution with diethanolamine. The mono- and di-substituted products were obtained simultaneously in fair to moderate yields via the acylation reaction (Scheme 2).

To determine the optimal reaction conditions for the efficient preparation of the target molecules, the reaction temperature was varied. The results indicated that room temperature is crucial for preventing the facile generation of anhydride during the addition of aroyl chloride. The target molecular structure depended on the molar ratio of compound **BNI** and aroyl chloride. When the molar ratio of compound **BNI** and aroyl chloride was less than 1:1.5, the product was primarily the mono-substitution product **4**. However, when the molar ratio was 1:2.2–1:2.5, both mono- and di-substituted products were generated. The yields of compounds **3** and **4** indicated that the substituents on the benzene ring exerted negligible effects. All of the 1,8-naphthalimide derivatives were confirmed via ^1^H-NMR, ^13^C-NMR, MS, elemental analysis and the data are reported in the experimental section.

**Figure 1:**
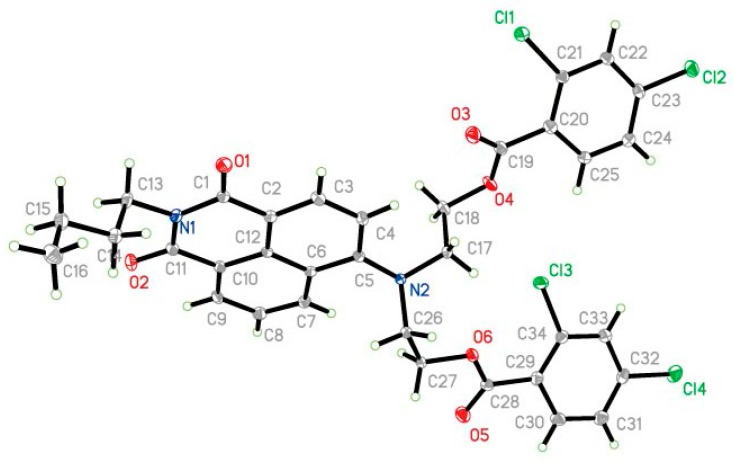
Molecular structure of **3f**.

**Figure 2:**
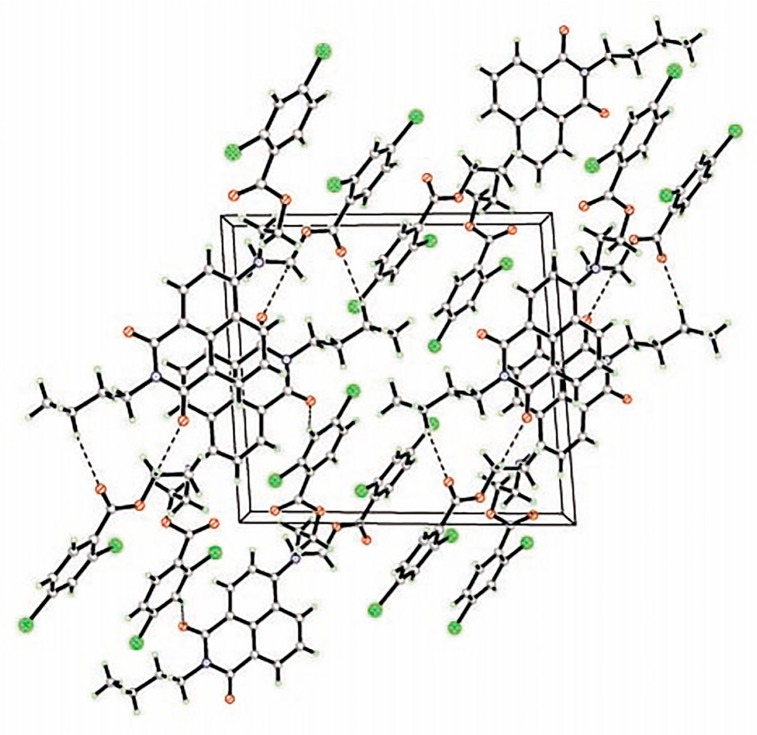
Packing view of **3f**.

**Figure 3:**
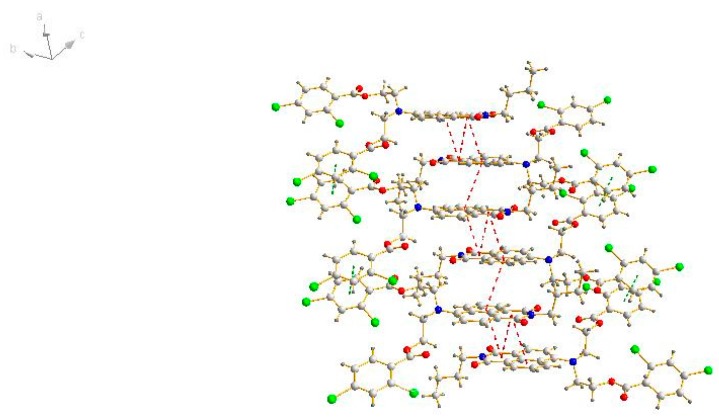
π-π Stacking interactions between the core planes.

To further confirm the structure of the synthesized compounds, a single-crystal structure of compound **3f** was obtained by dissolving the crystal in ethanol followed by slow evaporation of the solvent at room temperature over approximately 3 days. The molecular structure of compound **3f** is shown in Figure 1. A packing diagram is shown in Figure 2. In Figure 2 showed that all of the atoms of the rings were almost in the same plane. The 3D net consists of moderate π-π stacking interactions with a centroid-to-centroid distance of 3.6548 Å and the shortest distance (X1A to C2, C3) between the core planes was 3.490 Å (Figure 3).

### 2.2. Absorption Spectra and Fluorescence

The photo-physical properties of 1,8-naphthalimides mainly originate from the polarization of the naphthalimide moiety due to the electron donor-acceptor interaction that occurs between the substituents at ***C***-4 (electron donor) and the carbonyl groups from the imide structure (electron acceptor) of the molecule [29]. The normalized UV-vis spectra of compounds **3a**–**3f** and **4a**–**4f** in EtOH/H_2_O (*v*/*v* = 4:1) with a concentration of 1 × 10^−5^ M are listed in Table 1. As shown in Table 1, the main absorption band of these dyes is centered between 414 and 440 nm. The main absorption band of mono-substituted compounds was centered at 440 nm and that of the di-substituted compounds was centered at 420 nm because the substituents at benzene affected the molecular polarities of compounds **3** and **4**.

The emission was detected at 521–529 nm. Compared with the emission maximum of compound **3a** (*λ*_em_ = 521 nm), those of the other di-substituted compounds, namely **3b**–**3f**, were all slightly red-shifted, which might be the result of the electron-withdrawing and electron-donating capabilities of the substituents of the benzene. In addition, the emission maxima of the mono-substituted compounds were almost equal [30]. However, the mono-substituted compounds exhibited greater fluorescence quantum yield (*Φ*_F_) at approximately 329–398 nm, almost twofold greater than that of the di-substituted compounds. Di-substitution with hydroxyl groups, which exerted a steric effect, resulted in a bathochromic shift in the emission maximum. Because of this spatial steric effect, the fluorescence emissions of all the mono-substituted compounds exceeded those of the di-substituted compounds. This result indicated that the mono-substituted compounds might be better probe molecules due to their greater fluorescence quantum yield and the large space between the hydroxyl and carbonyl.

### 2.3. Solvent Effect

**Figure 4:**
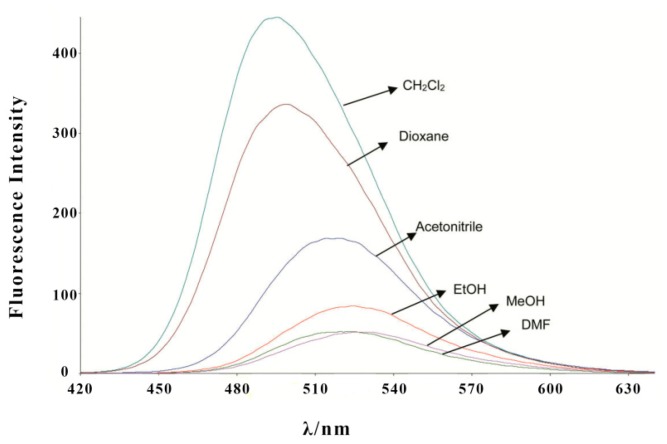
Various solvents’ influences on the fluorescence intensity.

Compound **3f** was selected for the fluorescence intensity experiment because its single crystal structure was determined by X-ray analysis, which was able to explain the ion-recognition performance based on the molecular configuration. As shown in Figure 4, the fluorescence emission of compound **3f** is more dependent on the solvent. As the solvent polarity increased, the emission wavelength *λ*_em_ was red-shifted and the fluorescence intensity decreased. The fluorescence maximum shifts to longer wavelengths from nonpolar to polar solvents are indicative of the charge transfer nature of the emitting state. As shown in Figure 4, the *λ*_em_ for **3f** shifted from 495 nm to 529 nm as the solvent was changed from dichloromethane to dimethylformamide. Examination of the chemical structure of entitled dyes suggested that the occurrence of an intramolecular charge transfer (ICT) process might be responsible for their solvent sensitivity. The absorption of light by these molecules is accompanied by electron density shift from the donor to the acceptor and most often, results in an increase in the dipole moment on going to the excited state. Depending on the mode of receptor attachment, binding of the cation would induce either a bathochromic or hypochromic shift of the absorption maximum with a simultaneous change in the intensity [31]. Therefore, EtOH and H_2_O were selected as the solvents to reduce the use of organic solvents and allow the dissolution of metal ions.

### 2.4. Selectivity of Probe ***3f***

**Figure 5:**
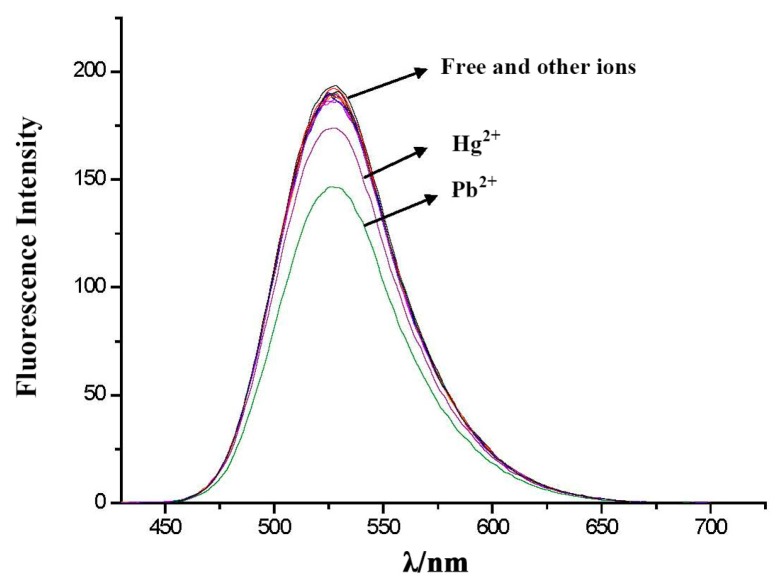
Fluorescence intensity response of probe **3f** to different metal cations.

**Figure 6:**
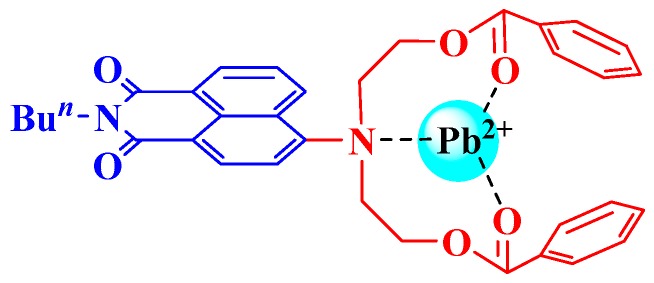
Proposed structure of the **3f**-Pb^2+^ complex.

Figure 5 shows the fluorescence emission spectra of compound **3f** in EtOH/H_2_O (*v*/*v* = 4:1) solution (1 × 10^−5^ M) that was excited at its absorption maximum. As shown in Figure 5, upon addition of 3 eq. of each cation, only Pb^2+^ induced a distinct spectrum change while other metal ions (Cu^2+^, Ag^+^, Na^+^, K^+^, Ba^2+^, Ca^2+^, Co^2+^, Mg^2+^, Mn^2+^, Ni^2+^, Sn^2+^, Zn^2+^, Al^3+^, Hg^2+^ and Fe^3+^) showed either no or slight changes in the fluorescence spectra relative to the free compound **3f**, which indicated that compound **3f** might be a probe molecule for Pb^2+^. The fluorescence intensity difference might be attributable to the formation of a compound **3f**-Pb^2+^ complex. The fluorescence intensity difference might be attributable to the formation of a **3f**-Pb^2+^ complex. The fluorescence intensity of **3f** is strong in original, after Pb^2+^ added, the N, carbonyl O and OH are likely to coordinate with it, resulting in fluorescence decrease. On the basis of the aforementioned fact, a plausible binding mode of the complex was proposed, as shown in Figure 6.

### 2.5. Titration Experiments

**Figure 7:**
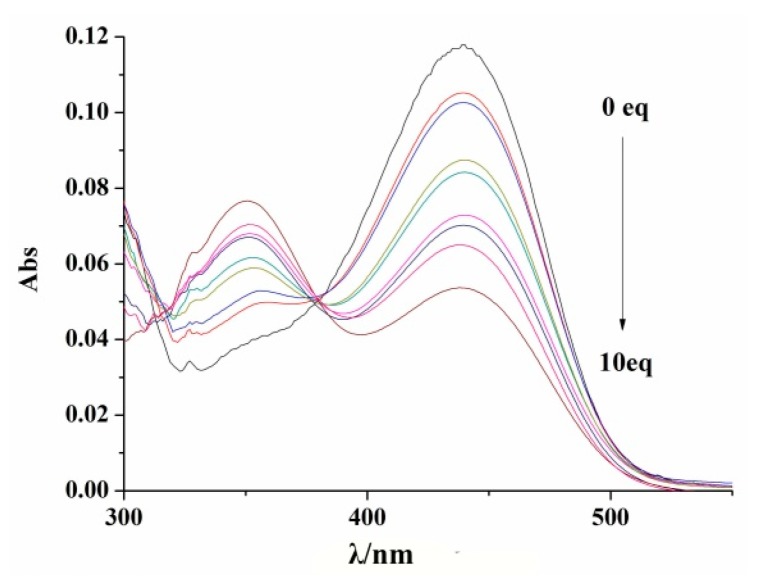
UV–vis spectra of probe **3f** under the same conditions except for the addition of different amount of Pb^2+^.

To further assess its utility as a Pb^2+^-selective probe, its UV–vis spectrum response to Pb^2+^ as demonstrated in Figure 7, in the concentration range of 0 and 10 eq. the absorbance of **3f** decreased from 0.118 to 0.054. The results led us to conclude that **3f** could be an effective colorimetric probe for Pb^2+^.

**Figure 8:**
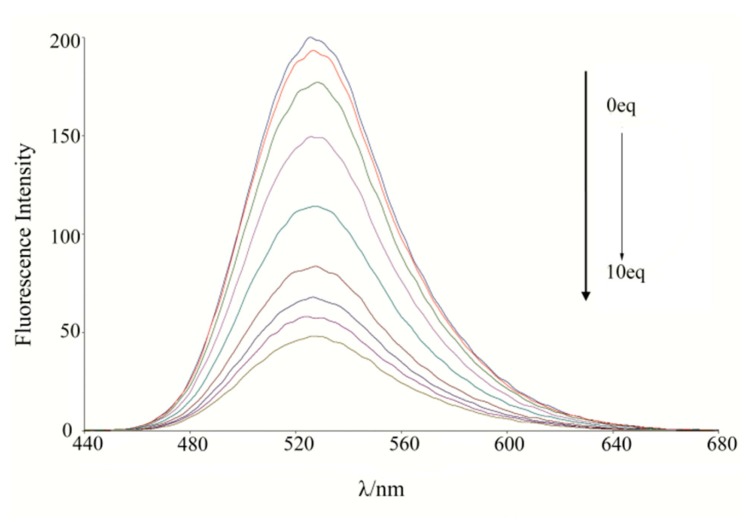
Fluorescence spectra of probe **3f** under the same conditions except for the addition of different amount of Pb^2+^.

As shown in Figure 8, in the concentration range of 0 and 10 eq., emission intensity decreased significantly with increasing Pb^2+^ concentration, implying that Pb^2+^ can be quantitatively detected in a wider concentration range. These results will support the design of novel fluorescent materials based on 1,8-naphthalimide derivatives.

### 2.6. Effect of pH

**Figure 9:**
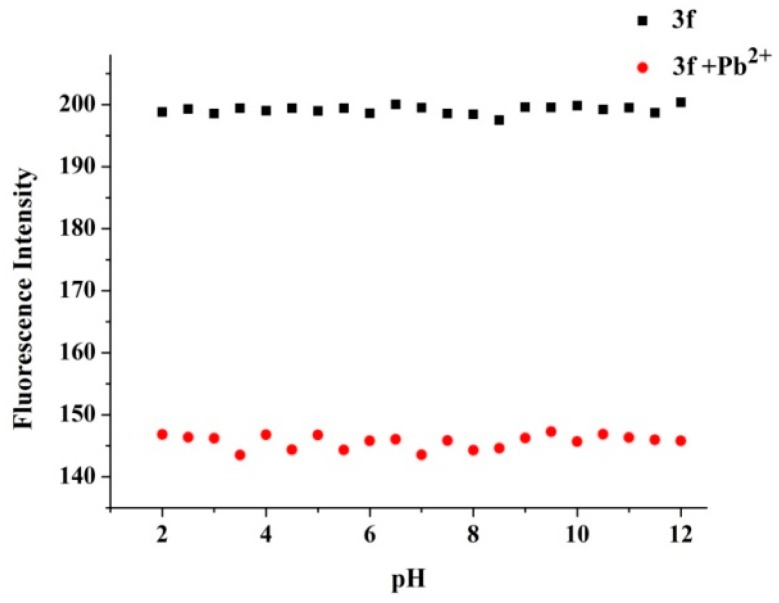
Effect of pH on the fluorescence intensity of **3f** in the absence or presence of 3 eq. Pb^2+^.

The pH effect on **3f** was investigated in EtOH-H_2_O solution (*v*/*v* = 4:1) in a pH value 2–12 (Figure 9). No obvious fluorescence emission change of probe **3f** was observed with the pH changes and this indicated that the free probe **3f** was stable at a wide pH range. Meanwhile, an obvious fluorescence decrease was observed after mixing **3f** with Pb^2+^ in the range pH 2–12. These suggested that compound **3f** could act as a fluorescent probe for Pb^2+^ under physiological pH conditions and also be applicable at complex environment conditions.

## 3. Experimental

### 3.1. Chemicals and Instruments

All of the solvents and reactants are commercially available and were used without purification. The melting points were determined using a Beijing Taike melting point apparatus (X-4) and were uncorrected. The ^1^H-NMR and ^13^C-NMR spectra were recorded on a Bruker AVANCE 300 MHz or 400 MHz nuclear magnetic resonance spectrometer using CDCl_3_ or DMSO-*d*_6_ as the solvent and TMS as the internal standard. The mass spectra were recorded on a Waters XevoTQ mass spectrometer. The elemental analysis was performed on FLASH EA1112 elemental analyzer. Absorption spectra were collected using a PERSEE TU-1900 ultraviolet spectrophotometer. Fluorescence emission spectra were obtained on a Perkin Elmer LS55 fluorospectrophotometer. The X-ray data were collected on a Bruker AXSII CCD area-detector diffractometer using graphite monochromated Mo Ka radiation (λ = 0.71073 Å) at 293(2) K. All of the calculations were performed with the SHELX-97 program package [32,33].

### 3.2. Testing Methods

Compound **3f** was dissolved in EtOH to form a 10^−^^4^ M stock solution. Deionized water was used throughout the experiment and the solutions of metal ions were prepared from CuCl_2_·2H_2_O, AgNO_3_, NaCl, KCl, BaCl_2_, CaCl_2_, CoCl_2_, MgCl_2_, MnCl_2_, NiCl_2_, Pb(NO_3_)_2_, SnCl_2_, ZnCl_2_, AlCl_3_, Hg(OAc)_2_ and FeCl_3_, respectively. All the cations were dissolved in deionized water to obtain 10^−2^ M stock solutions. For fluorescent measurement of Pb^2+^, the mixed stock solutions were diluted to 10 mL with EtOH and H_2_O to form EtOH/H_2_O (*v*/*v* = 4:1) solutions. For the titration experiments, **3f** stock solution (10 μL) was mixed with a certain amount of Pb^2+^ stock solution and diluted to 10 mL with EtOH and H_2_O to form EtOH/H_2_O (*v*/*v* = 4:1) solutions. The wide pH range solutions were prepared by adjustment of 0.05 mol·L^−1^ Tris-HCl solution with HCl or NaOH solution.

### 3.3. Synthesis of N-n-Butyl-4-bromo-1,8-naphthalimide *(**1**)*

*N*-n-Butyl-4-bromo-1,8-naphthalimide was prepared by modifying a previously reported procedure [24] to obtain an improved yield of 84%. 4-Bromo-1,8-naphthalic anhydride (58 mmol, 16.1 g) and n-butylamine (60 mmol, 4.4 g) were heated under reflux in ethanol (250 mL) with vigorous stirring for 12 h under N_2_. Then, the mixture was cooled and the precipitated solids were filtered and recrystallized from ethanol to yield 13.5 g (70%) of a light-yellow product. m.p. 105–106 °C; IR (KBr, $\bar{v}$ cm^−1^): 2932, 2848 (C-H) 1686 (C=O). ^1^H-NMR (DMSO-*d*_6_, 300 MHz), *δ* (ppm): 7.92–8.52 (m, 5H), 3.98–4.03 (t, *J* = 7.3 Hz, 2H), 1.56–1.66 (m, 2H), 1.31–1.39 (m, 2H), 0.90–0.95 (t, *J* = 7.3Hz, 3H). ^13^C-NMR (100 MHz, DMSO-*d*_6_) *δ* (ppm) 163.21, 132.95, 131.94, 131.94, 131.73, 131.73, 131.32, 131.32, 129.51, 128.60, 123.09, 122.31, 39.76, 30.01, 20.27, 14.18. MS (ESI) *m/z*: 332 (M + H)^+^. Anal. Calcd. For C_16_H_14_BrNO_2_ (%): C, 57.85; H, 4.25; N, 4.22. Found: C, 57.80; H, 4.18; N, 4.28.

### 3.4. Synthesis of N-n-Butyl-4-N′,N′-dihydroxyethyl-1,8-naphthalimide *(**BNI**)*

*N*-n-Butyl-4-(*N′*,*N′*-dihydroxyethyl)amino-1,8-naphthalimide was obtained using a procedure similar to that reported by Guo et al. [25]. *N*-*n*-Butyl-4-bromine-1,8-naphthalimide (45.2 mmol, 15 g) and diethanolamine (75 mL mmol) were mixed in ethylene glycol monomethyl ether (100 mL). The mixture was refluxed for 6 h. The crude product was purified by column chromatography using silica gel, with an EtOAc and light petroleum (*v*/*v* = 1:4) solution as the eluent. *N*-n-Butyl-4-(*N*′,*N*′-dihydroxyethyl)amino-1,8-naphthalene imide (**BNI**) was obtained as a yellow solid at a yield of 20.4%. m.p. 129–130 °C; IR (KBr, $\bar{v}$ cm^−1^): 3432 (O-H), 2957, 2872 (C-H) 1658 (C=O). ^1^H-NMR (400 MHz, CDCl_3_) *δ* (ppm) 8.88–7.28 (m, 5H), 4.16–4.12 (t, *J* = 7.2 Hz, 2H), 3.85–3.88 (t, *J* = 5.2 Hz, 4H), 3.61–3.64 (t, *J* = 5.2 Hz, 4H), 2.81 (s, 2H), 1.68–1.69 (m, 2H), 1.43–1.45 (m, 2H), 0.96–0.99 (t, *J* = 7.2 Hz, 3H). ^13^C-NMR (100 MHz, CDCl_3_) *δ* (ppm) 164.34, 163.91, 154.28, 131.18, 131.22, 130.85, 130.14, 127.24, 125.65, 122.92, 117.18, 117.05, 59.73, 59.73, 55.33, 55.33, 40.10, 30.20, 20.38, 13.85. MS (ESI) *m/z*: 357 (M + H)^+^. Anal*.* Calcd. for C_20_H_24_N_2_O_4_ (%): C 67.40; H 6.79; N 7.86. Found: C 67.28; H 6.86; N 7.95.

### 3.5. General Procedure for the Synthesis of N-n-Butyl-4-[N′,N′-dihydroxyethyl]-1,8-naphthalene Imide

A mixture of **BNI** (1.0 g, 2.81 mmol) and TEA (0.63 g, 6.25 mmol) solution in dichloromethane (40 mL) was stirred and substituted benzoyl chloride (6.25 mmol) was then slowly added over 30 min and allowed to react for 8 h at 25 °C. The product was neutralized by saturated Na_2_CO_3_(aq) and purified by column chromatography on a silica gel column using cyclohexane-EtOAc (*v*/*v* = 1:3) as the eluent.

#### 3.5.1. [(2-Butyl-1,3-dioxo-2,3-dihydro-1H-benzo[*de*]isoquinolin-6-yl)imino]diethane-2,1-diyl dibenzoate (**3a**)

Yield 46.0%, yellow solid, m.p. 148–150 °C; IR (KBr, $\bar{v}$/cm^−1^) 2960–2897 (C-H), 1719 (C=O). ^1^H-NMR (400 MHz, CDCl_3_) *δ* (ppm) 8.53–7.28 (m, 15H), 4.54–4.51 (t, *J* = 5.2 Hz, 4H), 4.17–4.13 (t, *J* = 7.2 Hz, 2H), 3.91–3.88 (t, *J* = 5.2 Hz, 4H), 1.75–1.67 (m, 2H), 1.50–1.41 (m, 2H), 1.01–0.97 (t, *J* = 7.8 Hz, 3H). ^13^C-NMR (100 MHz, CDCl_3_) *δ* (ppm) 166.15, 166.15, 164.28, 163.64, 153.96, 153.96, 133.13, 133.13, 131.74, 131.19, 130.26, 129.93, 129.51, 129.42, 128.27, 128.27, 128.27, 128.27, 127.61, 127.61, 125.92, 125.92, 123.92, 123.20, 118.85, 117.69, 62.06, 62.06, 52.76, 52.76, 40.03, 30.24, 20.39, 13.88. MS (ESI) *m/z*: 565 (M + H)^+^. Anal. Calcd. for C_34_H_32_N_2_O_6_ (%): C 72.32; H 5.71; N 4.96. Found: C 72.44; H 5.68; N 4.82.

#### 3.5.2. [(2-Butyl-1,3-dioxo-2,3-dihydro-1H-benzo[*de*]isoquinolin-6-yl)imino]diethane-2,1-diyl bis(2-methylbenzoate) (**3b**)

Yield 48.2%, yellow solid, m.p. 94–95 °C; IR (KBr, $\bar{v}$/cm^−1^) 2959–2871 (C-H), 1717 (C=O). ^1^H-NMR (300 MHz, CDCl_3_) *δ* (ppm) 8.54~7.08 (m, 13H) 4.51–4.47 (t, *J* = 5.4 Hz, 4H), 4.17–4.12 (t, *J* = 7.5 Hz, 2H), 3.96–3.87 (t, *J* = 5.4 Hz, 4H), 2.46 (s, 6H), 1.74–1.66 (m, 2H), 1.49–1.42 (m, 2H), 1.02–0.97 (t, 3H). ^13^C-NMR (75 MHz, CDCl_3_) *δ* (ppm) 166.96, 166.96, 164.38, 163.72, 153.96, 140.52, 140.52, 132.28, 132.28, 131.76, 131.76, 131.25, 130.47, 130.47, 130.31, 129.99, 128.68, 128.68, 127.55, 125.92, 125.92, 125.59, 125.59, 123.27, 118.70, 117.67, 61.68, 61.68, 52.83, 52.83, 40.12, 30.30, 21.75, 21.75, 20.46, 13.93. Anal. Calcd. for C_36_H_36_N_2_O_6_ (%): C 72.95; H 6.12; N 4.73. Found: C 72.98; H 6.21; N 4.78. 

#### 3.5.3. [(2-Butyl-1,3-dioxo-2,3-dihydro-1H-benzo[*de*]isoquinolin-6-yl)imino]diethane-2,1-diyl bis(4-methylbenzoate) (**3c**)

Yield 30.4%, yellow solid, m.p. 104–105 °C; IR (KBr, $\bar{v}$/cm^−1^) 2957–2927 (C-H), 1707 (C=O). ^1^H-NMR (400 MHz, CDCl_3_) *δ* (ppm) 8.56–7.13 (m, 13H), 4.53–4.51 (t, *J* = 5.6 Hz, 2H), 4.20–4.17 (t, *J* = 7.6 Hz, 2H), 3.91–3.88 (t, *J* = 5.2 Hz, 2H), 3.57–3.56 (m, 1H), 3.31–3.30 (m, 1H), 2.45–2.38 (m, 8H), 1.76–1.75 (m, 1H), 1.74–1.39 (m, 7H). ^13^C-NMR (100 MHz, CDCl_3_) *δ* (ppm) 171.88, 171.88, 166.32, 164.41, 163.76, 154.15, 154.15, 144.49, 143.91, 139.17, 134.15, 134.15, 130.24, 130.24, 130.24, 130.24, 129.51, 129.51, 129.19, 129.19, 129.01, 129.01, 126.76, 126.76, 126.41, 118.84, 61.94, 61.94, 52.79, 52.79, 40.09, 30.33, 21.76, 21.76, 20.42, 13.89. MS (ESI) *m/z*: 593 (M + H)^+^. Anal*.* Calcd. for C_36_H_36_N_2_O_6_ (%): C 72.95; H 6.12; N 4.73. Found: C 72.79; H 6.25; N 4.82.

#### 3.5.4. [(2-Butyl-1,3-dioxo-2,3-dihydro-1H-benzo[*de*]isoquinolin-6-yl)imino]diethane-2,1-diyl bis(4-chlorobenzoate) (**3d**)

Yield 33.7%, yellow solid, m.p. 88–90 °C; IR (KBr, $\bar{v}$/cm^−1^) 2957-2871 (C-H), 1718, 1690, 1664 (C=O). ^1^H-NMR (400 MHz, CDCl_3_) *δ* (ppm) 8.56–7.28 (m, 13H) 4.52–4.49 (t, *J* = 5.4 Hz, 4H), 4.19–4.11 (m, 2H), 3.88–3.84 (t, *J* = 5.4 Hz, 4H), 1.74–1.68 (m, 2H), 1.51–1.41 (m, 2H), 1.01–0.96 (t, *J* = 7.2 Hz, 3H). ^13^C-NMR (100 MHz, CDCl_3_) *δ* (ppm) 165.35, 164.25, 163.62, 153.73, 139.80, 139.80, 131.72, 131.35, 130.82, 130.82, 130.82, 130.82, 130.09, 129.97, 128.71, 128.71, 128.71, 128.71, 127.93, 127.93, 127.70, 126.09, 126.09, 123.34, 118.90, 117.99, 62.25, 62.25, 52.82, 52.82, 40.21, 30.33, 20.46, 13.91. MS (ESI) *m/z*: 633 (M + H)^+^. Anal*.* Calcd. for C_34_H_30_Cl_2_N_2_O_6_ (%): C 64.46; H 4.77; N 4.42. Found: C 64.55; H 4.71; N 4.38.

#### 3.5.5. [(2-Butyl-1,3-dioxo-2,3-dihydro-1H-benzo[*de*]isoquinolin-6-yl)imino]diethane-2,1-diyl bis[4-(trifluoromethyl)benzoate] (**3e**)

Yield 20.8%, yellow solid, m.p. 100–101 °C; IR (KBr, $\bar{v}$/cm^−1^) 2960-2926 (C-H), 1731 (C=O). ^1^H-NMR (400 MHz, CDCl_3_) *δ* (ppm) 8.58–8.46 (m, 2H), 7.91–7.89 (t, *J* = 4.0 Hz, 3H), 7.62–7.48 (m, 5H), 4.58–4.55 (t, *J* = 5.6 Hz, 3H), 4.18–4.15 (t, *J* = 7.6 Hz, 2H), 3.92–3.89 (t, *J* = 5.2 Hz, 3H), 1.49–1.28 (m, 4H), 1.01–0.98 (m, 8H). ^13^C-NMR (100 MHz, CDCl_3_) *δ* (ppm) 164.97, 164.97, 164.13, 163.54, 163.54, 153.51, 153.51, 134.89, 134.57, 132.70, 132.70, 131.65, 131.65, 131.37, 129.82, 129.82, 129.82, 129.82, 127.79, 126.14, 126.14, 125.38, 125.38, 125.34, 125.34, 123.39, 119.01, 118.20, 62.54, 62.54, 52.82, 52.82, 40.11, 30.21, 20.39, 13.80. MS (ESI) *m/z*: 701 (M + H)^+^. Anal*.* Calcd. for C_36_H_30_F_6_N_2_O_6_ (%): C 61.71; H 4.32; N 4.00. Found: C 61.65; H 4.44; N 4.08.

#### 3.5.6. [(2-Butyl-1,3-dioxo-2,3-dihydro-1H-benzo[*de*]isoquinolin-6-yl)imino]diethane-2,1-diyl bis(2,4-dichlorobenzoate) (**3f**)

Yield, 65.0%, yellow solid, m.p. 87–88 °C; IR (KBr, $\bar{v}$/cm^−1^): 2961-2866 (C-H), 1731, 1710, 1652 (C=O). ^1^H-NMR (300 MHz, CDCl_3_) *δ* (ppm) 8.58–7.15 (m, 11H) 4.54–4.5 (t, *J* = 5.4 Hz, 4H), 4.20–4.15 (t, *J* = 7.5 Hz, 2H), 3.91–3.87 (t, *J* = 5.4 Hz, 4H), 1.77–1.67 (m, 2H), 1.50–1.42 (m, 2H), 1.02–0.97 (m, *J* = 7.2 Hz, 3H). ^13^C-NMR (100 MHz, CDCl_3_) *δ* (ppm) 164.28, 164.28, 163.61, 163.61, 153.58, 138.80, 135.05, 135.05, 132.44, 132.44, 132.44, 131.67, 131.39, 131.15, 131.15, 131.15, 130.12, 129.95, 12.95, 127.58, 127.40, 126.92, 126.15, 123.34, 118.82, 117.95, 62.57, 62.57, 52.62, 52.62, 40.19, 30.33, 20.45, 13.91. MS (ESI) *m/z*: 703 (M + H)^+^. Anal*.* Calcd. for C_34_H_28_Cl_4_N_2_O_6_ (%): C 58.14; H 4.02; N 3.99. Found: C 58.26; H 3.95; N 3.89.

#### 3.5.7. 2-[(2-Butyl-1,3-dioxo-2,3-dihydro-1H-benzo[*de*]isoquinolin-6-yl)-(2-hydroxy-ethyl)-amino]-ethyl Ester (**4a**)

Yield 61.4%, yellow solid, m.p. 158–159 °C; IR (KBr, $\bar{v}$/cm^−1^) 3396 (O-H), 2965-2820 (C-H), 1720 (C=O). ^1^H-NMR (400 MHz, CDCl_3_) *δ* (ppm) 8.55–7.45 (m, 10H), 6.71 (d, *J* = 8.4 Hz, 1H), 6.01 (s, 1H), 4.78 (t, *J* = 10.4 Hz, 2H), 4.14 (t, *J* = 11.2 Hz, 4H), 3.78 (t, *J* = 10.4 Hz, 2H), 2.03 (s, 2H), 1.69 (t, *J* = 15.2 Hz, 2H), 1.24 (s, 2H), 0.95 (t, *J* = 14.8 Hz, 3H). ^13^C-NMR (100 MHz, CDCl_3_) *δ* (ppm) 167.83, 164.64, 164.13, 164.13, 149.04, 149.04, 134.24, 133.62, 131.14, 131.14, 129.81, 129.35, 128.58, 128.58, 126.07, 125.03, 123.19, 120.41, 111.06, 63.17, 60.40, 60.16, 43.98, 40.02, 30.33, 20.45, 13.89. MS (ESI) *m/z*: 461 (M + H)^+^. Anal*.* Calcd. for C_27_H_28_N_2_O_5_ (%): C 70.42; H 6.13; N 6.08. Found: C 70.34; H 6.25; N 6.03.

#### 3.5.8. 2-[(2-Butyl-1,3-dioxo-2,3-dihydro-1H-benzo[*de*]isoquinolin-6-yl)(2-hydroxyethyl)amino]ethyl-2-methylbenzoate (**4b**)

Yield 57.6%, yellow solid, m.p. 179–180 °C; IR (KBr, $\bar{v}$/cm^−1^) 3367 (OH), 2957-2927 (C-H), 1717 (C=O). ^1^H-NMR (400 MHz, CDCl_3_) *δ* (ppm) 8.60–7.37 (m, 9H), 6.76–6.74 (d, *J* = 8.0 Hz, 1H), 5.31 (s, 1H), 4.80 (t, 2H), 4.18–4.16 (t, *J* = 6.8 Hz, 4H), 3.81 (t, 2H), 2.42 (d, *J* = 8.4 Hz, 3H), 1.73–1.71 (d, *J* = 6.8 Hz, 2H), 1.47–1.45 (m, 2H), 1.27 (t, 3H), 0.98 (t, 3H). ^13^C-NMR (100 MHz, CDCl_3_) *δ* (ppm) 171.66, 168.02, 164.68, 164.17, 149.13, 138.44, 138.29, 134.48, 131.16, 131.16, 130.69, 128.37, 126.95, 126.17, 125.00, 123.16, 120.42, 110.98, 104.11, 63.12, 43.96, 43.96, 40.04, 30.33, 29.72, 21.27, 20.45, 13.90. MS (ESI) *m/z*: 475 (M + H)^+^. Anal*.* Calcd. for C_28_H_30_N_2_O_5_ (%): C 70.87; H 6.37; N 5.90. Found: C 70.75; H 6.44; N 5.92.

#### 3.5.9. 2-[(2-Butyl-1,3-dioxo-2,3-dihydro-1H-benzo[*de*]isoquinolin-6-yl)(2-hydroxyethyl)amino]ethyl-4-methylbenzoate (**4c**)

Yield 61.2%, yellow solid, m.p. 168–169 °C; IR (KBr, $\bar{v}$/cm^−1^) 3362 (O-H), 2957-2825 (C-H), 1704 (C=O). ^1^H-NMR (400 MHz, CDCl_3_) *δ* (ppm) 8.59–7.25 (m, 9H), 4.81–4.78 (t, *J* = 5.2 Hz, 2H), 4.20–4.13 (t, *J* = 7.6 Hz, 3H), 3.81–3.79 (t, *J* = 5.2 Hz, 2H), 2.45–2.43 (d, *J* = 8.8 Hz, 3H), 2.07 (s, 1H), 1.75–1.74 (m, 2H), 1.45 (m, 2H, CH_2_), 0.95 (t, *J* = 14.8 Hz, 3H). ^13^C-NMR (100 MHz, CDCl_3_) *δ* (ppm) 171.28, 167.94, 164.67, 164.17, 149.13, 144.49, 134.30, 131.14, 131.14, 130.23, 130.23, 129.85, 129.29, 126.15, 125.00, 123.16, 120.41, 110.96, 104.06, 63.01, 60.42, 60.42, 44.03, 40.03, 30.37, 21.72, 20.46, 13.90. MS (ESI) *m/z*: 475 (M + H)^+^. Anal*.* Calcd. for C_28_H_30_N_2_O_5_ (%): C 70.87; H 6.37; N 5.90. Found: C 70.72; H 6.48; N 5.95.

#### 3.5.10. 2-[(2-Butyl-1,3-dioxo-2,3-dihydro-1H-benzo[*de*]isoquinolin-6-yl)(2-hydroxyethyl)amino]ethyl-4-chlorobenzoate (**4d**)

Yield 60.5%, yellow solid, m.p. 167–168 °C; IR (KBr, $\bar{v}$/cm^−1^) 3361 (O-H), 2958-2927 (C-H), 1720 (C=O). ^1^H-NMR (400 MHz, CDCl_3_) *δ* (ppm) 8.53–7.41 (m, 9H), 6.71–6.69 (d, *J* = 8.4 Hz, 1H), 5.98 (s, 1H), 4.77–4.75 (t, *J* = 4.8 Hz, 2H), 4.14–4.11 (t, *J* = 8.4 Hz, 4H), 3.80–3.77 (t, *J* = 5.2 Hz, 2H), 2.03 (s, 1H), 1.70 (m, 2H), 1.42 (m, 2H), 0.95–0.91 (t, *J* = 6.4 Hz, 3H). ^13^C-NMR (100 MHz, CDCl_3_) *δ* (ppm) 166.84, 164.59, 164.08, 164.08, 149.00, 140.13, 134.18, 131.14, 131.14, 129.68, 128.92, 128.92, 127.78, 126.05, 125.01, 123.17, 120.40, 111.07, 104.08, 63.39, 60.41, 43.80, 40.02, 30.33, 20.45, 14.22, 13.89. MS (ESI) *m/z*: 451 ((M + H)-CH_3_CH_2_OH)^+^. Anal*.* Calcd. for C_27_H_27_ClN_2_O_5_ (%): C 65.52; H 5.50; N 5.66. Found: C 65.59; H 5.42; N 5.72.

#### 3.5.11. 2-[(2-Butyl-1,3-dioxo-2,3-dihydro-1H-benzo[*de*]isoquinolin-6-yl)(2-hydroxyethyl)amino]ethyl-4-(trifluoromethyl)benzoate (**4e**)

Yield 50.4%, yellow solid, m.p. 161–162 °C; IR (KBr, $\bar{v}$/cm^−1^) 3389 (O-H), 2886–2775 (C-H), 1715 (C=O). ^1^H-NMR (400 MHz, CDCl_3_) *δ* (ppm) 8.56–7.62 (m, 9H), 6.75–6.73 (d, *J* = 4.8 Hz, 1H), 5.96–5.95 (m, 1H), 4.82–4.80 (t, *J* = 4.8 Hz, 2H), 4.16–4.12 (t, *J* = 7.2 Hz, 4H), 3.84–3.81 (t, *J* = 5.2 Hz, 2H), 2.03 (s, 1H), 1.71–1.67 (m, 2H), 1.45–1.39 (m, 2H), 1.26 (s, 1H), 0.97–0.93 (t, *J* = 7.2 Hz, 3H). ^13^C-NMR (100 MHz, CDCl_3_) *δ* (ppm) 166.47, 164.58, 164.09, 164.09, 148.98, 134.17, 133.70, 133.14, 131.22, 131.12, 131.12, 129.69, 129.40, 126.82, 126.13, 125.00, 123.16, 120.47, 111.14, 104.20, 63.48, 63.48, 63.48, 43.39, 40.01, 30.34, 20.45, 13.90. MS (ESI) *m/z*: 529 (M + H)^+^. Anal*.* Calcd. for C_28_H_27_F_3_N_2_O_5_ (%): C 63.63; H 5.15; N 5.30. Found: C 63.74; H 5.20; N 5.19.

#### 3.5.12. 2-[(2-Butyl-1,3-dioxo-2,3-dihydro-1H-benzo[*de*]isoquinolin-6-yl)(2-hydroxyethyl)amino]ethyl-2,4-dichlorobenzoate (**4f**)

Yield 62.3%, yellow solid, m.p. 178–179 °C; IR (KBr, $\bar{v}$/cm^−1^) 3377(O-H), 2959–2929 (C-H), 1725 (C=O). ^1^H-NMR (300 MHz, CDCl_3_) *δ* (ppm) 8.63–6.76 (m, 8H), 4.83–4.79 (t, *J* = 4.5 Hz, 2H), 4.20–4.15 (t, *J* = 7.5 Hz, 2H), 3.85–3.82 (t, *J* = 4.8 Hz, 2H), 2.07–2.06 (s, 1H), 1.73–1.72 (m, 2H), 1.49–1.42 (m, 2H), 1.27(s, 3H), 1.00–0.96 (t, *J* = 4.5 Hz, 3H), 0.86 (m, 1H). ^13^C-NMR (75 MHz, CDCl_3_) *δ* (ppm) 165.78, 164.61, 164.11, 148.78, 139.14, 134.94, 134.18, 132.77, 131.24, 131.22, 129.73, 127.56, 127.31, 125.91, 125.14, 123.32, 120.47, 111.42, 104.25, 63.66, 43.42, 40.04, 40.40, 30.32, 29.72, 20.45, 13.90. Anal*.* Calcd. for C_27_H_26_Cl_2_N_2_O_5_ (%): C 61.25; H 4.95; N 5.29. Found: C 61.28; H 4.90; N 5.25.

The spectra of synthesized compounds are available in supplementary material.

## 4. Conclusions

A series of novel *N*-n-butyl-1,8-naphthalimide derivatives with mono- and di-substitution at position 4 were synthesized via direct arylation. Probe **3f** exhibited an obvious quenched fluorescence in the presence of Pb^2+^ over a range of metal cations. A titration of monomer with Pb^2+^ ion was performed. When Pb^2+^ ion concentration increased from 0 to 10 eq., the fluorescent intensity of **3f** decreased from 199.97 to 48.21 and the absorbance of **3f** decreased from 0.118 to 0.054. The results led us to conclude that **3f** could be an effective colorimetric and fluorescent probe for Pb^2+^. In addition, the pH effect on **3f** showed that this sensor should be valuable for Pb^2+^ analysis in environmental samples and the biological systems. 

## Figures and Tables

**Table 1:** Absorption and fluorescence characteristics of compounds **a**–**f** in EtOH/H_2_O (*v*/*v* = 4:1) solution.

Comp.	Log *ε* (1 M^−1^·cm^−1^) ^a^	*λ*max ^b^	*A*	*λ*em ^b^	*Φ*_F_^c^
**3a**	4.286	421.5	0.103	521	0.264
**3b**	3.748	419.5	0.090	529	0.245
**3c**	4.100	420.0	0.035	524	0.289
**3d**	3.699	414.0	0.120	522	0.238
**3e**	4.336	417.0	0.055	527	0.238
**3f**	4.017	421.5	0.118	528	0.247
**4a**	4.241	439.5	0.228	526	0.554
**4b**	4.255	439.5	0.121	524	0.596
**4c**	4.233	440.5	0.054	523	0.577
**4d**	4.140	440.0	0.168	523	0.600
**4e**	4.201	440.0	0.116	522	0.579
**4f**	4.212	438.5	0.098	521	0.599

^a^ Extinction coefficient. ^b^ Maximum absorbance (*λ*_abs_) and emission intensity (*λ*_em_) wavelengths. ^c^ Fluorescence quantum yield (*Φ*_F_) were determined by using rhodamine 6G in an EtOH solution. (*Φ*_F_ = 0.94) as a reference.

## Supplementary Materials

The supplementary materials are available online.
